# Integrating *in vitro* and *in silico* approaches for exploring antidiabetic potential of dimethyl and thiomethyl indolinone derivatives

**DOI:** 10.1371/journal.pone.0319987

**Published:** 2025-05-16

**Authors:** Kashif Ali, Ahmed A. Elhenawy, Waqas Alam, Khalaf F. Alsharif, Khalid J. Alzahrani, Haroon Khan, Long Chiau Ming, Rahul G. Ingle

**Affiliations:** 1 Department of Pharmacy, Abdul Wali Khan University Mardan, Mardan, Pakistan; 2 Chemistry Department, Faculty of Science, Al-Azhar University, Nasr City, Cairo, Egypt; 3 Department of Clinical Laboratory Science, College of Applied Medical Science, Taif University, Taif, Saudi Arabia; 4 Department of Pharmacy, Korea University, Sejong, South Korea; 5 Faculty of Medical and Life Sciences, Sunway University, Sunway City, Malaysia; 6 Datta Meghe College of Pharmacy, Datta Meghe Institute of Higher Education and Research (Deemed to be University), Wardha, India; Kafrelsheikh University Faculty of Pharmacy, EGYPT

## Abstract

Diabetes mellitus (DM) is a metabolic disorder caused by insulin deficiency. It is a rapidly growing problem with immense social, economic and health related problems. The market available drugs for the management of DM possess several limitations and therefore the discovery of more effective agents is the need of time. The current study was designed to evaluate two oxindole derivatives: (*E*)-3-(2,4-Dimethylbenzylidene)-6-chloroindolin-2-one (compound **1**), and (*E*)-3-(4-(Methylthio) benzylidene)-6-chloroindolin-2-one (compound **2**) against α-amylase, protein glycation, and DPP-IV. In case of *in vitro* antidiabetic activities, compound **1** demonstrated significant inhibition at various concentrations with IC_50_ value of 32.917 µg/mL against α-amylase, 210.592 µg/mL against protein glycation and 31.28 µg/mL against DPP-IV. Similarly, when tested at various concentrations, compound **2** illustrated marked inhibition with IC_50_ value of 42.691 µg/mL on α- amylase, 341.551 µg/mL on protein glycation and 47.192 µg/mL against DPP-IV. Molecular docking studies identified the binding patterns of oxindoles in the active site of targeted protein and enzymes. Moreover, the docking analysis also showed the potent interaction of test compounds with the target sites against α-amylase, protein glycation and DPP-IV. In conclusion, both the compounds are significant inhibitors of α-amylase, protein glycation and DPP-IV *in vitro* assays with the support of molecular dynamic studies and therefore are potential candidates for further studies.

## 1. Introduction

Diabetes mellitus (DM), a chronic metabolic disorder caused by the ineffective production of insulin by pancreas [1,2]. DM is the main cause of prolonged ill health and early mortality. It is very fatal than HIV-AIDS with nearly 01 death every 10 seconds [3]. The islet cells of the pancreatic islets of Langerhans create insulin in response to the blood glucose level. At the cellular level, insulin interacts with an insulin receptor, a protein found on the cell surface. The role of 3 PPAR- in preventing vascular tissue from being harmed by high blood glucose levels has also been demonstrated. This effect is unrelated to how it affects insulin resistance or blood glucose regulation [4].

The rise in the prevalence of diabetes mellitus in most areas across the globe has been linked with economic development, leading to expansion and adoption of modern lifestyle habits [5].

Oxindoles (1, 3-dihydro-2 H-indole-2-ones), are organic heterocyclic compounds containing a benzene ring attached with the pyrrole ring bearing carbonyl group at 2^nd^ position. These compounds are categorized as hetero-aromatic organic compounds frequently encountered in mammalian tissues, bodily fluids, and diverse plant species [6]. These privileged skeletons are broadly found in the pharmaceutical compounds and natural products [7]. Oxindoles exhibited an extensive range of biological activities such as anti-diabetic [8], anti-cancer [9,10], anti-HIV [11], antitumor [12], kinase inhibitory [13], anti-Alzheimer [14], neuro-protective [15], and analgesic activity [16].

In the current study, the selected oxindole derivatives are studied for possible anti-diabetic effects using the *in vitro* assays including DPP-IV, protein glycation, and α-amylase. Moreover, these compounds underwent molecular docking screening to determine their binding potentials in the corresponding protein pockets against α -amylase and DPP-IV inhibitory test).

## 2. Materials and methods

### 2.1 *In vitro* antidiabetic activity

#### 2.1.1 α-Amylase inhibitor.

The standard protocol of *in vitro* α-amylase inhibition was performed according to Naeem et al. 70 µL of a 50 mM phosphate buffer (pH 6.0) were combined with 100 µL of the reaction solution. 10 mL (0.057 units) of an enzyme, and 50 µL of the compound being examined was added to the solution. After being thoroughly blended, a 10-minute pre-incubation at 25 °C was carried out. The absorbance of the resulting mixture was measured at a wavelength of 400 nm. Further, a solution equal to 10 µL a 0.5 mM concentration of p-nitrophenyl glucopyranoside was added to the solution, and re-incubation carried out at a 25 °C temperature. The absorbance of the solution was measured at a wavelength of 400 nm after incubation [17].

#### 2.1.2 Protein glycation assay.

Briefly, 50 µl of protein solution in distil water was added to 10 µl of glyceraldehyde solution in black microplates. The plates were sealed and incubated at 37 ^o^C for 24 h. After incubation, test compound solutions were then added. Blank containing distilled water served as a negative control (untreated), while Rutin as a positive control. The fluorescence was measured at 370 nm (excitation), and 440 nm (emission) [18]. The percentage inhibition was calculated using the following formula:


$$Percentinhibition=1-\frac{fluorescenceoftestwell}{fluoresecenceofnegativecontrol}x100$$

#### 2.1.3 DPP-IV inhibition assay.

The assay was carried out according to the established method with slight modification [19]. Various concentrations of the synthesized compounds were added to human recombinant DPP-IV enzyme solution in respective wells of a 96-well plate. After incubation, pNA substrate (Gly-Pro-pNA) dissolved in Tris buffer was added to initiate the reaction and further incubated. After incubation, acetic acid solution was added to stop the reaction and the absorbance was recorded. The percentage inhibition was calculated by using the following equation:


$$Percentinhibition=1-\left(\frac{absorbanceofthetestwell}{absorbanceoftheuntreatedcontrol}\right)x100$$

The IC_50_ values were calculated with Graph pad prism software to find the concentrations of the synthesized compounds at which 50% inhibition can be achieved.

### 2.2 Molecular docking

Molecular docking was performed to explore the binding patterns of compounds in the active site of different target receptors. Chem Office Ultra 2006 was used for the purpose of creating the two-dimensional structures of all inhibitors. The protein was prepared in MOE software with default parameters which included protonation, energy minimization, and deletion of nonpolar hydrogens. Protein Data Bank (PDB) identifiers: **1B2Y**, **4IW2**, and **8FF7**. The preliminary Acarbose, α-D-Glucopyranose, and Ascorbic acid—serving as inhibitors for α-amylase, protein glycation, and peroxidase, respectively redocked into the crystal frameworks. Furthermore, the efficacious performance of the targeted compounds was authenticated *via* the low values of RMSD (3.20 Å(**1B2Y**), 2.41 Å for (**4IW2**), and 2.19 Å (**8FF7**) which were acquired through the root mean square deviation between the native and redocked poses of the co-crystallized inhibitors. All ligands including standard inhibitor was protonated and energy minimized before the docking protocol was implemented. All ligands were saved as (mdb file format) which was to be used further for docking. The entire ligand database was docked into the protein’s binding region. To validating the docking methodology, ensuring that the predicted interactions are consistent with known binding modes. The Ligand-Protein complex was created in ten different conformations, each of which had a different docking score. The correctness and efficacy of the docking technique were checked numerous times by repeating the docking method. After each complex had its two-dimensional and three-dimensional images taken, it was evaluated for different kinds of interactions. Molecular docking of the selected compounds was performed using PyRx-Autodock wizard as the engine for docking to explore ligand-protein interactions [20].

### 2.3. MD simulation

Based on the docking data, the best pose of α-glucosidase–4b complex was selected for further examination. The complex was subjected to a molecular dynamics analysis using the CABS-iMODs module which provides an enhanced normal mode analysis (NMA) approach in inner coordinates at a constant temperature of 300 K and constant pressure of 1 atm [21]. Finally, the target complex was simulated using molecular dynamics for 100 ns.

## 3. Results

### 3.1. α-Amylase inhibitory effect

**Fig 1:**
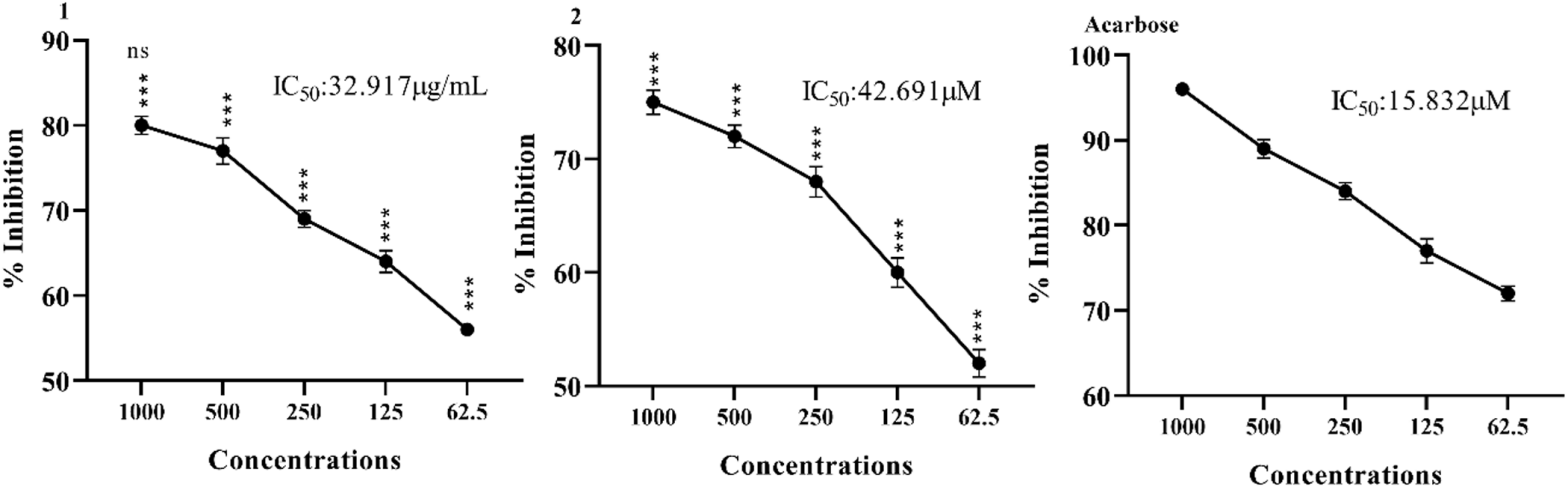
*In vitro* α-amylase inhibitory effect of oxindole derivatives. Results were expressed as Mean ± SEM of 03 independent values. One-way ANOVA and Bonferroni were applied to the values. Significance was determined at p < 0.005, p < 0.01, p < 0.001 compared with positive control (Acarbose) at the same doses.

The *in vitro* α-amylase inhibitory effect of tested oxindole derivatives is represented in Fig 1. The compounds elicited significant inhibition at various concentrations. The half maximal concentration of standard drug acarbose was 15.832 μg/mL. The compound **1** exhibited significant inhibition with maximum inhibition of 80% at 1000 μg/mL while IC_50_ value of was 32.917 μg/mL. Similarly, compound **2** illustrated α-amylase inhibition at test concentrations with maximum inhibition of 75% at 1000 μg/mL and IC_50_ value was calculated as 42.69 μg/mL.

### 3.2. *In vitro* protein glycation inhibitory effect

**Fig 2:**
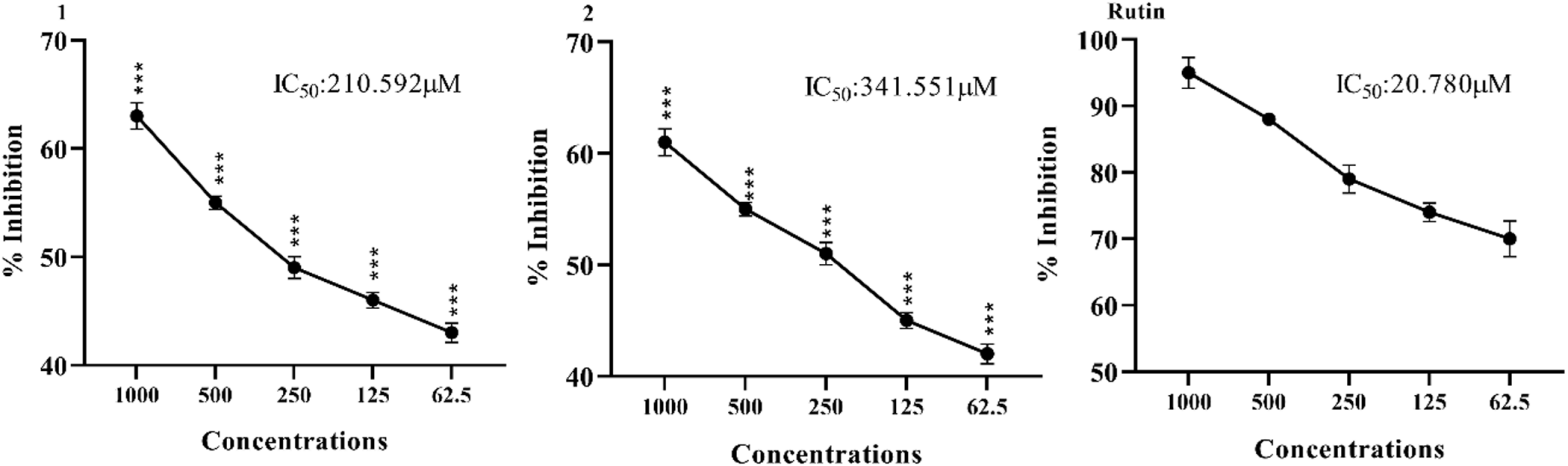
*In vitro* protein glycation inhibitory effect of oxindole derivatives. Results were expressed as Mean ± SEM of 03 independent values. One way ANOVA and Bonferroni was applied to the values. Significance was determined at p < 0.005, p < 0.01, p < 0.001 compared with positive control (Rutin) at the same doses.

Fig 2 summarizes the *in vitro* protein glycation inhibitory effect. The highest percent inhibition was exhibited by standard drug Rutin which was 20.780 μg/mL. Amongst the oxindole derivatives compound 1 inhibited the activity of protein glycation by 210.592 μg/mL while compound 2 inhibited activity 341.551 μg/mL as shown in Fig 2.

### 3.3. *In vitro* DPP-IV inhibitory effect

**Fig 3:**
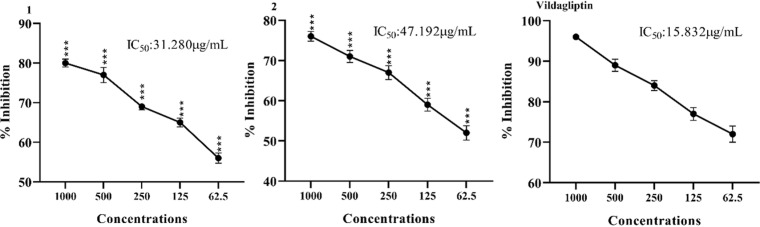
*In vitro* DPP-IV inhibitory effects of oxindole derivatives. Results were expressed as Mean ± SEM of 03 independent values. One way ANOVA and Bonferroni was applied to the values. Significance was determined at p < 0.005, p < 0.01, p < 0.001 compared with positive control (Rutin) at the same doses.

The *in vitro* DPP-IV inhibitory effect of the synthesized oxindole derivatives is shown in Fig 3. The highest percent inhibition was exhibited by standard drug vildagliptin which was 15.832 µg/ml. Amongst oxindole derivatives compound 1 inhibited the activity of DPP-IV by 31.280 µg/ml while compound 2 inhibited DPP-IV activity by 47.192 µg/ml, as shown in Fig 3.

### 3.4. Docking of oxindoles

#### 3.4.1. Binding free energies.

The binding free energies (ΔG) of tested compounds are presented in (Table 1). The initial inhibitors have been introduced into their binding sites sufficiently to reach their crystal configurations. The RMSD showed that the binding pocket was more stable in the lowest score positions. The data was utilized to rank the docked poses and identify the most effective docked conformation for each compound. the selected docking conformations of **1** and **2** compounds exhibited the most suitable configuration inside the active sites. The primary active sites of 1B2Y including Glu233, Asp300, Asp197, Gly306, and His201 are known to interact with the inhibitor a standard α-amylase inhibitor, acarbose. Similar, the enzyme with PDB code 4IW2 interacts through its active site (Tyr150, His242, and Arg222) with α-D-glucopyranose, a type of glucose that is important for the metabolism of carbohydrates. Moreover, His163 is the active site of protein with PDB code 8FF7 strongly interacts with ascorbic acid, a known standard antioxidant. The results displayed that the compounds **1** and **2** interacted with significant residues in the binding pocket of these enzymes. The inhibitory behavior of both compounds is described in term of binding energy (BE) with the receptor. After that, these compounds were redocked, and the results were compared to the respective reference inhibitors, and obtained a root mean square deviation (RMSD) as presented in (Table 1).

**Table 1:** The binding-affinity for compounds 1 and 2 with docking score (kcal/mol).

	ΔG	rmsd	E._Int_	E._H.B_
1B2Y				
Compound 1	-5.67	1.20	-16.87	-9.20
Compound 2	-5.63	1.75	-22.46	-8.98
Acarbose	-5.71	1.72	-25.23	-11.57
4IW2				
Compound 1	-9.21	1.01	33.53	-8.70
Compound 2	-6.65	1.61	38.07	-11.67
*α-D-Glucopyranose*	-8.67	1.35	43.16	-10.07
8FF7				
Compound 1	-5.670	1.198	-16.866	-9.201
Compound 2	-5.634	2.745	-22.462	-8.979
Ascorbic	-4.938	1.133	-12.590	-10.315

Where, *ΔG*: Free binding energy of the ligand; RMSD: *root-mean-square deviation; H*.B.: H-bonding energy between protein and ligand; EInt.: Binding affinity of H-bond interaction with receptor.

#### 3.4.2. Binding interaction against 1B2Y as α-amylase inhibitor.

**Fig 4:**
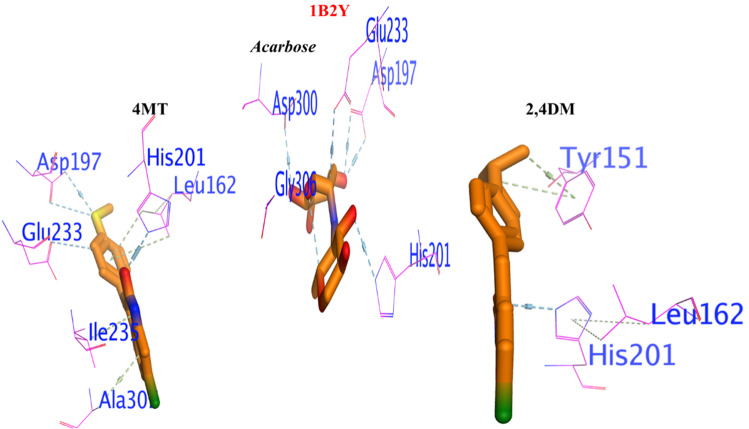
3D docking poses of the 1 and 2 compounds against 1B2Y.

The compounds **1** and **2** exhibited significant binding affinity including (ΔG = -5.67 Kcal/mol., and ΔG = -5.63 Kcal/mol.) compared to acarbose (ΔG = -5.71 Kcal/mol). As shown in Fig 4, compound **1** binds to the enzyme’s active site and develops important interactions with important residues, including a hydrogen bond between the nitrogen atom of chlorooxindole and HIS201 and a π-π contact between Tyr151 and the 2,4-dimethylphenyl ring. Tyrosine is a hydrophilic aromatic amino acid, which means that the delocalized electrons of the chloroxindole nucleus in its benzene ring have non-polar attractions, further support the potential of **1** to stabilize the enzyme-substrate complex. Since the chlorine atom and the active site residues are located close, it is also believed that they can stabilize the chemical connection between the amino acids in the active site. Similarly, compound **2** formation of hydrogen bonds with Glu233, His201, and Asp197, and arene-arene interactions with Leu162, Ile235, and Ala300, align with the binding mode of acarbose, reinforcing the validity of our findings. This revealed that both **1** and **2** could be promising candidates as α-amylase inhibitors.

#### 3.4.3. Binding interaction against 4IW2 as protein glycation inhibitor.

**Fig 5:**
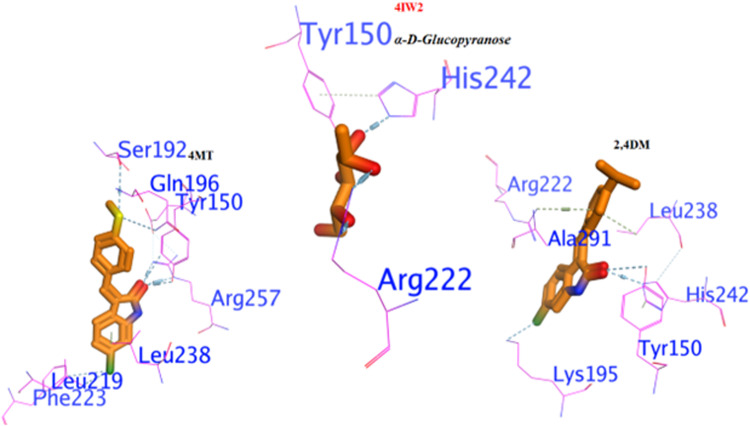
3D docking poses of the compounds 1 and 2 compounds against 4IW2.

The two tested compounds (**1** and **2**) exhibited significant potency against protein glycation enzyme compared to α-D-Glucopyranose, which was used as a standard as shown in Fig 5. The molecular docking analysis showed that compound **1** had a more negative binding free energy (ΔG = -9.21 Kcal/mol.) than α-D-Glucopyranose (ΔG = -8.67 Kcal/mol), and holding the active site by forming a strong H-bond with HIS242, Lys195, and Tyr150, also, a π−H interaction with Leu238, Arg222, and Ala291. Similarly, compound **2** exhibited lower inhibition than the standard with binding free energies of ΔG = --6.65 Kcal/mol. its interacted with the enzyme similarly to the reference inhibitor, forming key hydrogen bonds with ARG257, TYR150, Gln195, Leu219, and Phe223 and Ser192.

#### 3.4.4. Binding interaction against 8FF7 as DPP4 inhibitor.

**Fig 6:**
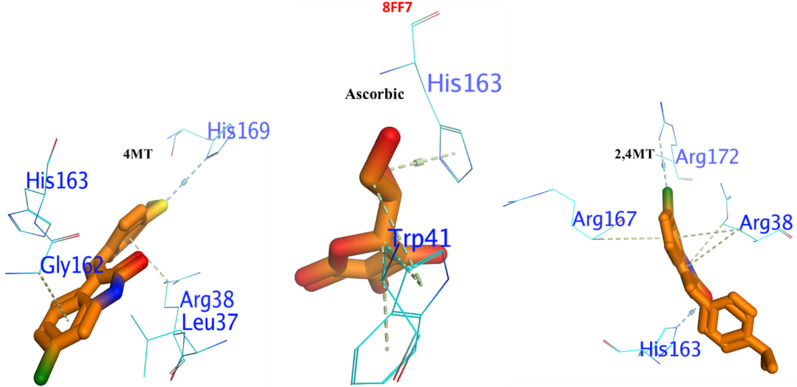
Binding interaction of the compounds 1 and 2 compounds against 4FF7 as DPP4 inhibitor.

As shown in Fig 6, the molecular interactions indicate that the target proteins and active compounds **1** and **2** illustrate a strong binding affinity. The hydrogen bonds with Arg172 and His163 for compound 1 reflect a strong and specific attachment within the hydrophilic binding pocket. It was augmented by the n-π interactions with Arg38 and 167. Similarly, in case of compound **2**, the docking in the 4FF7 binding pocket through interactions with His169 and the formation of n-π interactions with Arg38 and Gly162 further supported the binding’s specificity.

In 3D-molecular docking, the parallel orientation of both compounds containing His163 may indicate the optimal alignment for interaction. The insights gained from these exchanges can guide structural alterations that enhance the stability, efficacy, and specificity of potential treatment choices.

#### 3.4.5. Molecular dynamic simulation against α-amylase.

**Fig 7:**
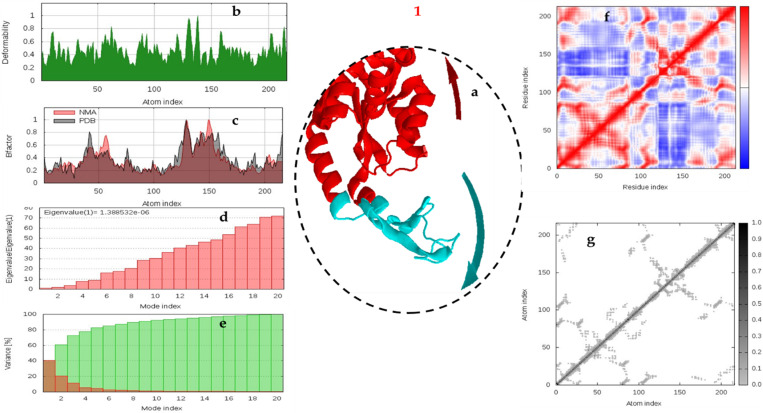
Multifaceted Analysis of MD trajectories for or 1– α-amylase. protein at 0-100 ns simulation time. Molecular mobility evaluated by NMA of which the two colored afﬁne arrows display the mobility or the direction of motion, where the longer arrows indicate greater motion, **b**) deformability, **c**) B-factor plot; **d**) eigenvalue, **e**) and variance plot; **f**) elastic network model; and **g**) covariance map.

**Fig 8:**
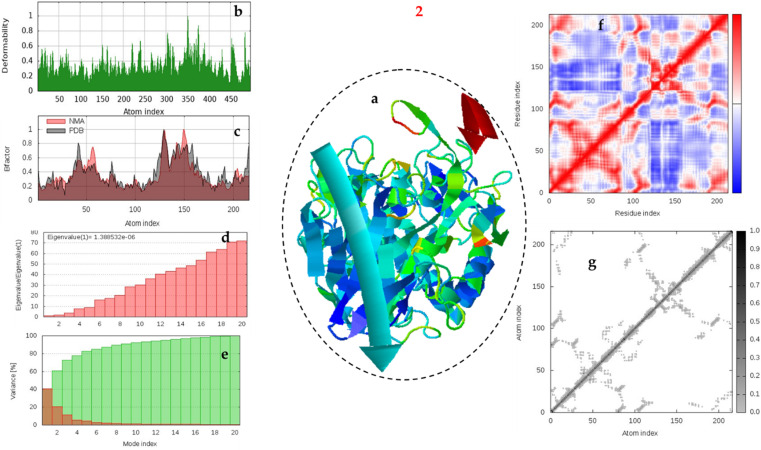
Multifaceted Analysis of MD trajectories for 2– α-amylase. protein at 0-100 ns simulation time. a) Molecular mobility evaluated by NMA of which the two colored afﬁne arrows display the mobility or the direction of motion, where the longer arrows indicate greater motion, b) deformability, c) B-factor plot; d) eigenvalue, e) and variance plot; f) elastic network model; and g) covariance map.

The normal mode analysis (NMA) and molecular dynamics (MD) simulations were utilized to provide insights into the structural flexibility and dynamic behavior of α-amylase, which are critical for drug design and the comprehension of various biological processes (Figs 7 and 8). MD simulation complements NMA by providing a time-resolved view of protein dynamics under physiological conditions. By simulating the motion of atoms over time, MD offers insights into the transient states of the protein and the interactions with ligands like **1, and 2**.

The 1-amylase appears to have a more pronounced peak in deformability around atom index 150, suggesting a highly flexible region. On the other hand, 2-amylase complex shows a more gradual increase in deformability towards the higher atom indices, indicating a more distributed flexibility (Figs 7b and 8b). The deformability plots of the 1-amylase and 2-amylase complexes provide insightful contrasts in their structural dynamics. The 1-amylase complex, with its significant fluctuations and prominent peaks in deformability, indicates a structure primed for flexibility. This inherent adaptability could be pivotal for its enzymatic functions, allowing it to accommodate a diverse array of substrates through conformational changes. In contrast, the 2-amylase complex’s more subdued deformability profile suggests a structure optimized for stability rather than flexibility. This rigidity may limit its substrate versatility but could enhance its resilience against environmental stresses, potentially making it a more reliable catalyst under consistent conditions. The distinct deformability characteristics of these complexes underscore the intricate balance between flexibility and rigidity that is crucial for their biological roles. While the 1-amylase complex may excel in catalytic versatility, that may be explain the higher activity. The B-factor, also known as the Debye-Waller factor or temperature factor, is a critical parameter in structural biology techniques that reflects the degree of atomic motion within a protein structure. A higher B-factor indicates increased atomic displacement, which can be interpreted as regions of the protein with greater flexibility. This flexibility often correlates with functionally important sites, such as active sites where substrate binding and catalysis occur, or protein-protein interaction interfaces. The comparison between the 1-amylase and 2-amylase complexes is intriguing, as it suggests distinct dynamic properties that could be related to their functional mechanisms or stability (Figs 7c and 8c). The 1-amylase complex, with its significant fluctuations and peaks in the B-factor plot, may have regions that are more adaptable to binding substrates or undergoing conformational changes essential for its activity. On the other hand, the 2-amylase complex appears to be more structurally constrained, which might reflect a different mode of action or interaction with other molecules. Understanding these differences at the atomic level can provide valuable insights into the molecular basis of enzyme function and regulation.

The eigenvalues derived from principal component analysis (PCA) are indeed critical indicators of motion within molecular structures. In the context of enzyme complexes like amylases, these eigenvalues can reveal how the conformational dynamics relate to their catalytic functions. The steep incline observed in the eigenvalue plot for the 1-amylase complex suggests a high degree of flexibility, which may be essential for its enzymatic activity, allowing it to adapt to different substrates or react to environmental changes (Figs 7d and 8d). Conversely, the more modest incline for the 2-amylase complex points to a more stable, perhaps more energy-efficient structure, which could imply a different mode of substrate interaction or a variation in its biological role. Understanding these dynamic properties is crucial for designing inhibitors or modifying enzymes for industrial applications.

Variance plots are a crucial tool in structural biology, providing insights into the dynamic behavior of proteins. In the case of the 1-amylase complex, the rapid decrease in variance after the initial peak suggests that a small number of modes are responsible for most of the dynamic activity. These dominant modes likely correspond to the essential movements required for the protein’s biological function (Figs 7e and 8e). On the other hand, the 2-amylase complex displays a more uniform variance across modes, implying a structure that is dynamically stable with no single mode dominating its motion. This could reflect a protein that operates through a collective harmony of movements rather than relying on a few key motions. Understanding these dynamics is key to deciphering the functional mechanisms of these proteins and can have significant implications in the design of drugs and enzymes.

The elastic network model (ENM) provides a simplified representation of the protein’s flexibility and rigidity. Regions with higher rigidity are crucial for maintaining structural integrity, while flexible regions are important for functional movements. The ENM for the 1-amylase complex shows a gradient from red (rigid) to blue (flexible), with several flexible regions (Figs 7f and 8f). These flexible regions are likely involved in substrate binding and catalytic activity, allowing the enzyme to adapt to different substrates and perform its function efficiently. The ENM for the 2-amylase complex also shows a gradient from red to blue, but with more extensive rigid regions compared to the 1-amylase complex. This suggests that the 2-amylase complex is generally more rigid, which could contribute to its structural stability and resilience under various conditions.

Covariance maps illustrate the correlated motions between residues within a protein. Darker regions indicate stronger correlations, suggesting coordinated movements that are crucial for the protein’s function. The covariance map for the 1-amylase complex shows several dark regions, indicating strong correlations between specific residues. These correlated motions suggest that certain regions within the 1-amylase complex move together, which could be important for its catalytic activity and substrate binding (Figs 7g and 8g). The covariance map for the 2-amylase complex displays fewer and lighter dark regions compared to the 1-amylase complex. This indicates weaker correlations between residues, suggesting that the 2-amylase complex has less coordinated motion and potentially more independent movements of its residues.

## 5. Discussion

One of the traditional risks for the onset of diabetes and its sequelae has been hyperglycemia. Therefore, maintaining blood glucose control is essential for both reducing macro- and microvascular problems and treating diabetes mellitus early. One treatment strategy is to stop the body from absorbing carbohydrates after eating, which is made possible by inhibiting the enteric enzymes α-glucosidase and α-amylase that are found in the intestine’s brush boundaries [22].

The synthesis of novel oxindole derivatives has been the subject of various research studies, which has laid the foundation for the identification of lead candidates in medical perception. The Spiro heterocyclic compounds having oxindole already reported for their potential α-amylase inhibitory activities [23]. Kanwal, et al., synthesized indoles and assessed them for α-amylase inhibition. All synthesized compounds showed potential inhibition against α-amylase [24]. Imran, S., et al., synthesized indole-based derivatives which exhibited moderate *α*-amylase inhibition activity [25]. Similarly, the synthetic compound, 3,3-di(indolyl)indolin-2-ones observed with α-amylase inhibitory activities. Overall, these compounds showed good α-amylase inhibitory activities [26]. Mohammad, B.D., et al., also synthesized and evaluated new 5-(2,2,2-trifluoroethoxy)phenyl-1,3,4-oxadiazol-2-thiol derivatives for their α-amylase inhibitory activities. Acarbose showed higher activity compared to these synthetic compounds [27].

Our results illustrated significant α-amylase inhibitory effects of test compounds under prescribed experimental conditions as shown in Fig 1. The activity of these electron donating oxindole derivatives can be attributed to the attached functional groups. Amylase inhibition activity of the synthesized compound **1** may be due to two methyl groups attached at the position 2 and 4 of the 6-chloro oxindoles. Amylase inhibition activity of our synthesized compound **2** could be attributed to methyl groups attached at the position 4 of the 6-chloro oxindoles.

Protein glycation is a multifaceted process of non-enzymatic covalent chemical interactions that transpire between the amino group of proteins and the carbonyl group of reducing sugars. Advance glycation end product generation and accumulation have been shown to be exacerbated by hyperglycemia, the clinical characteristic of poorly managed diabetes [28]. Khan et al., evaluated Oxindole-based chalcones analogues and they showed potent inhibitory activity against glycation compared to the reference Rutin [29]. Similarly, Isatin and its derivatives which showed different pharmacological activities including anticonvulsant, anti-HIV, anticancer, antiviral, antibacterial, antifungal, anti-tubercular, antiglycation, anti-inflammatory, analgesic, antimalarial, antioxidant, anthelmintic and antianxiety activity [30].

In the current research, *in vitro* protein glycation activity of electron donating oxindole derivatives were evaluated and it was found that both the compounds demonstrated potential inhibition against of protein glycation It can be attributed that *in vitro* protein glycation activity of electron donating oxindole derivatives might be due to the attached functional groups. Protein glycation inhibitory effect of the synthesized compound **1** is due to two methyl groups attached at the position 2 and 4 of the 6-chloro oxindoles. Protein glycation inhibition activity of the synthesized compound 2 is due to methyl groups attached at the position 4 of the 6-chloro oxindoles.

Dipeptidyl peptidase IV (DPP-IV) is essential for the regulation of blood sugar levels because it is involved in the digestion of incretin hormones [31]. Mohammad et al., witnessed that almost all derivatives were having substitutions with various heterocyclic compounds or alkyl groups. Many derivatives induced strong conformational changes to the DPP-IV enzyme, resulting in excellent anti-diabetic activity [27]. It is already reported that oxadiazole patents have grown up by almost 100% in the previous decade, reaching a total of almost 640, making this a very good compound in the community. They concluded that the oxadiazoles are potent enough to be established as impending antidiabetics more specifically as DPP-IV inhibitors [32]. Furthermore, these researchers concluded that the derivatives which passed the criteria were preceded for the wet lab synthesis and biological assessment. They concluded that various derivatives were potential compounds to be established as potent DPP-IV inhibitors [33]. *In vitro* DPP-IV inhibitory activity of electron donating oxindole derivatives can be endorsed by the attached functional groups. *In vitro* DPP-IV inhibitory effect of the synthesized compound **1** is due to two methyl groups attached at the position 2 and 4 of the 6-chloro oxindoles. *In vitro* DPP4 inhibitory effect activity of the synthesized compound **2** is due to methyl groups attached at the position 4 of the 6-chloro oxindoles.

The molecular docking of compounds **1** and **2** as potential α-amylase inhibitors, is a significant advancement in the search for new diabetes treatments. The reported ΔG values indicate a strong binding affinity, which is crucial for the effectiveness of an inhibitor. The ability of these compounds to form hydrogen bonds and π-π interactions with the active site of the enzyme suggests a stable interaction, which is essential for inhibitory activity. This stability, combined with the promising ΔG values, positions **1** and **2** as strong candidates for further research and development.

The research on chlorooxindoles is a significant stride in the quest for effective inhibitors against protein glycation enzymes. These enzymes a non-enzymatic process where reducing sugars react with amino groups on proteins, DNA, or lipids, can lead to the formation of advanced glycation end products (AGEs) with various pathophysiological implications. The synthesized chlorooxindoles not only showed promising potency but also demonstrated a stronger affinity to the active site of the enzyme compared to the standard α-D-Glucopyranose. The molecular docking analysis revealed that the **1** compound had a more negative binding free energy, suggesting a more stable complex formation with the enzyme. This compound’s ability to form strong hydrogen bonds and π-H interactions with key amino acids in the active site underscores its potential as a robust inhibitor. Although the second compound showed lower inhibition, its interaction pattern with the enzyme mimics that of the reference inhibitor, which could be advantageous in designing derivatives with improved efficacy.

The molecular docking suggested that the active compounds **1** and **2** exhibited a high affinity for binding with dipeptidyl peptidase enzymes. The specific interactions, such as hydrogen bonding with Arg172 and His163, as well as n-π interactions with Arg. 38 and 167 respectively, demonstrate a precise and robust connection within the enzyme’s hydrophilic binding site. This is further corroborated by the docking results of **2** in the binding pocket of the 4FF7 protein structure, where it forms interactions with His169 and establishes n-π interactions with Arg38 and Gly162. The parallel alignment of these compounds with His163 during the 3D-molecular docking suggests an optimal configuration for interaction, which is crucial for the efficacy of potential drug candidates. These findings provide valuable insights for the rational design of new compounds with our findings.

The comparison between the 1-amylase and 2-amylase complexes reveals distinct differences in their deformability profiles (Fig 1). The 1-amylase complex exhibits higher overall flexibility, which could be beneficial for catalytic versatility and interaction with a broader range of substrates. On the other hand, the 2-amylase complex’s increased rigidity might confer greater structural stability, making it more resilient under varying conditions. The higher flexibility of the 1-amylase complex suggests it may be more adaptable in its catalytic functions, potentially interacting with a wider variety of substrates. Conversely, the 2-amylase complex’s rigidity could imply a more specialized function with enhanced stability. The rigid regions in both complexes are essential for maintaining their structural integrity. However, the balance between flexibility and rigidity differs, reflecting their unique functional roles and stability requirements.

The comparative analysis of B-factor, eigenvalue, and variance plots highlights the differences in flexibility, stability, and dynamic behavior between the 1-amylase and 2-amylase complexes. Understanding these differences is crucial for elucidating their functional mechanisms and potential applications in biotechnology and medicine. The 1-amylase complex exhibits higher flexibility, as indicated by its higher B-factors and eigenvalues. This flexibility may enhance its catalytic versatility and ability to interact with various substrates. The 2-amylase complex is more rigid, with lower B-factors and eigenvalues. This rigidity may confer greater structural stability, making it more resilient under different conditions. The 1-amylase complex has dominant motions that contribute significantly to its dynamics, while the 2-amylase complex has a more even distribution of contributions from different modes, reflecting its stable structure.

The Analysis of ENM and Covariance map between the 1-amylase and 2-amylase complexes reveals distinct differences in their dynamic behavior and structural properties. The 1-amylase complex exhibits higher flexibility, as indicated by its covariance map and ENM. This flexibility may enhance its catalytic versatility and ability to interact with various substrates. In contrast, the 2-amylase complex is more rigid, which may confer greater structural stability. The stronger correlations in the 1-amylase complex suggest that its residues move in a more coordinated manner, which could be important for its enzymatic function. The weaker correlations in the 2-amylase complex indicate more independent movements, reflecting its stable structure.

## 6. Conclusion

In the current research, dimethyl and thiomethyl Indolinone derivatives (**1** and **2**) displayed significant inhibition against alpha amylase, protein glycation, and dipeptidyl peptidase-IV. Similarly, the molecular docking described the detailed interactions, particularly the hydrogen bonds, along with the n-π interactions, suggesting a strong and specific binding affinity. Such precise interactions within the hydrophilic binding site of the enzyme are indicative of a high potential for inhibitory activity. Molecular dynamics revealed the rigid regions in both complexes are essential for maintaining their structural integrity, and supported further the *in vitro* and docking studies.
